# On Edge

**Published:** 1892-05

**Authors:** C. M. Wright

**Affiliations:** Cincinnati, O.


### On Edge.

BY DR. C. M. WRIGHT, D.D.S., CINCINNATI, O.

Read before the Mississippi Valley Dental Society, March 9, 1892.

I have made a mistake in the selection of a subject, or title. The head-lines and the subject matter of my paper may not bear that harmonious correspondence one with the other, which is so gratifying to the logically inclined scientific mind. The mistake happened in this way, when I received an invitation from Dr. Custer to read a paper at this meeting, I accepted with a modest hyperæmia of the peripheral capillaries of my face and head. When I received a second invitation to name the paper—the unborn product of a secretion from my cortex cerebri—before I knew its sex or condition, when I had only promised to do my best with the means I had, to cause a paper to be born, I naturally felt as though I were "on edge", and I selected the subject nearest to my physical or mental condition.

I could so much more easily have written on some other subject. I might have called my paper "Confessions". Not of an opium eater, for De Quincy has made that a classic in English; nor of a religious fanatic, who argues the point of whether life is worth living, for Tolstoi has been before me in Russia, in French, and is living up to his doctrines. I should not have written the confessions of a double chloride of gold patient. The newspapers have done and are doing that. My confessions would have been simply the confessions of a dentist. An every day dentist. A " dollar a day " dentist. (I have left a space before the dollar, so that it can read a 20 dollar a day or a 100 dollar a day dentist, as you please). A dentist I mean who is occupied as we all are, in the " demnition grind " of life, such as Mantalini found at the mangle. A dentist who, when he dies can expect no greater praise, no more picturesque epitaph than the following:

" He never gained immortal fame,
Nor conquered earthly ills.
But men mourn for him just the same,
He always paid his bills."

The confessions of such a man I might have written, and you, my dear hearers, and you, the unnumbered host of readers who would perhaps see my confessions in print in the Dental Journals might have had the opportunity of seeking solitude to relieve the discharge of feeling and the flushing of lachrymal ducts that might follow. And the discharge and the flushing would do you good. You are all "on edge". You live in a continual strain, straining to suppress within the bounds of law and order, the law and order established by society and called politeness, your natural feelings.

You have, all day long in your offices, the disagreeable tingling sensations perceptibly creeping up every afferent nerve and down every efferent nerve in your bodies, the sensation defined by Bacon as resulting from bringing an acid in contact with the enamel of the teeth. You are conscious during every minute which you spend in your operating rooms that you are "on edge", on the edge of a yawning precipice. The precipice is not there any more than the acid is in contact with your enamel, but the sensation is, and you suppress it and maintain an outward appearance of placid dignity or interested kindliness, or tender sympathy. You are like the Spartan youths, who are reported to have looked steadily and with smiling denial on their faces, into the accusers' eyes, while the foxes concealed in their bosoms were gnawing at their vitals. Why have we these foxes gnawing at our vitals? Why are our "nerves" always "on edge?" It is because that for from eight to ten hours each day we are engaged in the performance of delicate and trying surgical operations. It is because some of our surgical operations require so much time and endurance that our nervous centres become exhausted from the long continued stimulation. It is because we must give so much of what psychologists define as "artificial attention", which is a very different article from the spontaneous attention of the child or the savage. This sort of attention is a cultivated effort on the part of the will, and it is a direct exercise of nervous force with a complicated inhibitory strain on a large number of motor nerves. It is because we have no diastolic recuperation. The systolic period is too long. It lasts all day. The general surgeon who begins with his knives and saws at 8 o'clock every morning and employs them upon one patient after another without rest until 5 o'clock in the afternoon, is "on edge," just as we are.

The mechanic who uses saws and knives in a shop, works ten hours each day with comparative ease, and does not understand what being "on edge" means. The dentist who works at the bench in his laboratory, no matter how delicate the skill demanding his labor may be, is not "on edge." He enjoys labor as an artist can, and he might solder and carve 12 hours a day and keep it up for fifty years without suffering nervous strain or exhaustion. But the man who adapts the appliance to the living tissues is engaged in an entirely different way. He is engaged in surgery, and surgery is "per se" an excitor of nerve centre activity. Mechanical operations are not. Surgery implies manual operations but it also implies healing, and our title of "D.D.S." implies the healing by manual operations certain lesions of the living body. The driving of a threaded needle through the lips of a gaping wound, and taking stitches to bring these lips together, and afterwards when living matter has succeeded in sealing the sides of a wound together by a sticky exudate and has sent out cells and buds to repair and regenerate the severed tissue, and re-instate the circulation of blood through repaired blood vessels, to remove the stitches is a very simple surgical operation, as far as the manual operation is concerned, it is very much like the tailor's work on a rent in one's garment, and the taking out of basting threads. But the tailor can sit cross legged on his table the live long day, and can sing and whistle, and never suspect that he has nerves or nerve centres, while the surgeon expends in fifteen minutes nervous force enough to run an entire tailoring establishment.

The surgeon is "on edge" delicately sharpened, keenly ground to a cutting edge, and the tailor is like a blunt hammer in comparison. There is no edge to be destroyed. The dental surgeon has this edge sharpened like a razor, and yet he hacks away for eight continuous hours each day, and expects to recuperate sufficiently each night. I need not detail the thousand and one things, tough things, hard things, that come in contact with this fine edge every hour in our daily practice. You who have been in practice for ten years, and who started in with a great supply of elasticity and resiliency have not begun to suffer much. You rather enjoy the kicking against the pricks. You who have gone around the track for another ten years, and reckon your dental experience as a twenty year one, begin to suspect that your work in life is extra hard. You who have had thirty years of this mill of the gods feel dead certain that it "grinds exceeding small." You are the men whose long and broad experience has taught you to entertain a colossal respect for the nervous system of man. You are the men who study with the deepest personal interest the physiology of nervous matter, and wonder at the correlated force that presides over every contraction of a muscle cell, that grasps with an intelligent hand every blood vessel, ready to respond in an instant to the slightest call for an increase of the life giving fluid to any tissue or organ or part, that maintains with never ceasing oscillation the heat producing and the heat reducing conditions of the tissues, so that the temperature is regulated more perfectly than by the delicate apparatus on the vulcanizers in our laboratories; that adjusts the supply to the demand for nourishment from every remote cell in the organism. This nervous system with its centres keenly sensitive to everything within and without the body, and ready to respond to every impression. You are the men who appreciate that this sensitive system cannot last forever, and that you are driving it to its utmost capacity in your daily work. You have held a tight rein upon these centres for thirty years in your daily and hourly operations in order that you might (unconsciously though it may be) influence by suggestion the nervous centres of all classes and conditions of minds as presented by your patients. In one day you will exercise this suggestion, at the expense of your own stock of physiological force, on old men and women, on boys and young girls, on children and hysterical mothers. After each case, be it more or less trying, you bob up serenely, and begin again the struggle. Your calm face does not tell the secret of your palpitating heart and your quivering nerves. Perhaps some of my hearers do not quite believe in the truth of the picture presented here. They may point to the pioneers in our profession who are still vigorous, intellectually and physically. We, of Cincinnati, well remember our departed friend, Dr. A. Berry, an octogenarian, and a man of unconquerable vitality. He it was who pooh poohed the idea of men becoming exhausted by eight or ten hours a day, and boasted that sixteen hours per day would be a fair day's work for a dental surgeon. He laughed to scorn the young man who complained of his nerves. But Dr. Berry was an exception to the rule among men. He had his own methods of relief from the strain of daily practice. I have studied him well. Firstly, Dr. Berry did a large share of laboratory work himself. This is a relief from surgery. Secondly, if I may be permitted to say, with deep respect for his memory, and as a friend and admirer, Dr. Berry constantly found vent for his overcharged nerve centres by vigorous invectives against what he considered wrongs and abuses, in other men's diet and morals, and religious doctrines. Why! The situation of our Garfield Monument in the middle of Race street must have added months if not years to Dr. Berry's nervous activity, for it furnished to him a constant escape valve for the compressed and surcharged nervous excitations caused by daily practice. His ever ready and picturesquely expressed anger at the idiocy of people who would permit a monument to be placed in the middle of a thoroughfare to frighten horses, certainly acted exactly like a steam valve well set to blow off when the pressure reached a certain point, and the boiler is saved from strain. The nervous system is so delicately *en rapport* with the entire world about us, and the most minute particle of matters within us, that the advanced students of physiology feel like babes playing on the sand with an unknown ocean of knowledge spread out before them, and just as the pebble is cast into this ocean by the child on the shore causes waves to circle about it and widen out to eternity, so every impression upon the nerve centres may cause ripples that will be felt in generations to come. Every time a child is spanked, a wave of trouble washes the control centres and the reflexes set every cell, or every spoke of living matter " on edge,"

and nutrition, function aud development are affected, favorably or unfavorably.

How do we know that the faults that we call congenital, let us say even in the enamel of the adult tooth, the weak spots that cannot resist the invader, the soil that proves suitable for pathogenetic micro-organisms were not made predisposing causes by faults in nutrition and that these faults in nutrition were owing to nervous impressions excited by pain, from the concussion of the mother's hand upon a sensitive nerve, situated at a very remote distance from the enamel forming organ of the child? Have you ever thought upon this aspect? Richter asks, "Have you, learned men, ever stopped to calculate the effect of a mother's scream on the character of the unborn child," and I ask, have you, wise dentists, ever stopped to calculate that the defective spots on the enamel might have been caused by the spanking received in babyhood?

Es ist nicht unmöglich.

---

DISCUSSION ON DR. C. N. WRIGHT'S PAPER.

*Dr. F. A. Hunter.*—Mr. President and Gentlemen of the Mississippi Valley Society: In the moment of weakness when I consented to open the discussion on Dr. Wright's paper, I must have been "on edge." To open a discussion on one of Wright's papers is a difficult matter, he usually goes so thoroughly into the subject that he leaves nothing for anybody else to say. The only escape left me is to differ from him. I can not differ in all points, but in some I think there is opportunity for differing. There is no doubt that a dentist in active practice is frequently worried and annoyed to a very great extent; but it is not confined alone to that profession; all have the same pecularity, a man in any profession who is busy, is necessarily "on edge." A man in the actual practice of dentistry is simply "in the swim or out of it." We can not control our practice to any considerable extent; if we have business to do we must attend to it and take the necessary annoyances and worries pertaining to it or we shall find ourselves not in it at all. I think if we look at the generality of the men in the dental profession, men who have been in practice thirty or forty years, such men as Taft, Morrison, Smith and myself, we shall see that they will compare favorably with men who have been busy in other professions the same length of time. All things considered, I think Dr. Wright places too much stress upon this point.

*Wm. Knight, M.D.*—I do not know that I have any remarks to make "on edge;" Dr. Wright has, in a very witty manner, told the truth that pertains to all professional men. I think however, that the surgeon or physician is "on edge" just the same as the dentist.

*Dr. Wright.*—Dr. Knight's remarks refer to all professions, I made a difference between dentists and those of Dr. Knight's profession. We have eight hours of continuous work without the rest between times that the physician frequently has in going to and fro to visit his patients; he has to visit a patient in one end of town, and another in another part of town, which is not like continuous work. The office hours of the physician are the most trying of the whole day, and that is just what the dentist has all the time. I think if they had a continuation of office hours instead of visiting, their profession would be as trying as that of the dentist. I admit that a surgeon is under a great strain when performing an operation, but the question is whether we do not have a stronger, harder strain caused by continuous work.

*Dr. H. A. Smith.*—Dr. Wright is right in many respects, when we look at the operator at the chair and consider the length of time he has to stand, there is a wide difference between his work and that of the physician who has patients at regular hours. The physician, after his office hours, has his recreation in getting in his carriage and going off to visit his patients. I have been in practice twenty-one years; in the first ten years of practice I did not mind it at all, but for the past five it has told on me and I have had to be careful in many ways, diet, etc. We just live, as it were, in a small place and work hard from morning until night and it does wear on us; it does tell on us; it tells on me and every one who has a good practice; but it is true that we must attend to our practice if we desire to keep it. We might look at the business man, he is "on edge," being pushed with business and if he can not get the money to meet his demands, certainly he is "on edge," but no comparison with the dentist; it is the length of time that the dentist has to stand at the chair that tells on his nervous system.

*Dr. Morrison.*—I am somewhat in the same condition as Dr. Hunter. The ground has been so well covered by our essayist that it leaves nothing for us to say. In the Mississippi Valley farther west we have the same little irritations and annoyances that seem to wear so upon the man; the only relief, I think, is to have something like the late Dr. Berry; we must all be cranks in some things, or at least have something outside our office to do to take off the nervousness and friction caused by continually standing at the chair, the "sharp edgedness" that Dr. Wright terms it. I have denominated this condition as an electrical one. There is often a want of proper electrical balance between our patients and ourselves, and it is also aggravated and increased by the direct contact of the instrument against the moist, softened condition of the dentine. When we can manage to keep the dentine thoroughly dry, it will be found that we can in a measure restrain this electricity and be comparatively comfortable. There is an infinite dynamo in the mouth. This is noticeable when we are using rubber polishing points, and the like; patients often complain of tickling of lip or nose. This is actually the case caused by electricity generated through friction of the rubber point. It is just the same to my mind as though caused by a small dynamo.

*M. H. Fletcher.*—It is proven beyond doubt that every organism is possessed of a certain amount of electricity, but the amount varies. Electricity is carried better by some individuals than others, and coming in contact with this class of patients, through the medium of an excavator or other instrument, we experience this feeling of being "on edge" to a greater degree. The great difficulty is in doing away with the induction currents. We have found nothing as yet to accomplish this. It does seem to me that through this medium we are in contact with this agent in spite of all we can do. When we attain a certain stage of life the tissues of the body do not repair as rapidly as they did formerly, and therefore we are not as able to withstand this influence, and it tells more readily on us; yet if we are able to control ourselves we can better control our patients. An abundant supply of fresh air and good light in the operating room is also a great aid. I think what Dr. Morrison has stated is founded upon facts, and there is an opportunity for investigating and experimenting in this direction.

*W. N. Morrison.*—Individually I have great relief from recreation. I fortunately possess a place in the country, and frequently take a run out there, freeing my mind entirely from my office and practice, and after working on the farm for one day I go back to my office feeling like a new man. There is a tendency among the profession to overwork; six or eight hours a day is long enough, and when your day's work is finished free your mind from business, and play the rest of the time, and you will find yourselves better off for so doing.

Dr. Smith also spoke of the necessity for some outside occupation in order to work off the nervous irritation, saying "all professional men work hard—lawyers, physicians and ministers—but the lawyer has the advantage of a long summer vacation; and who of us takes that? The minister gets a sore throat occasionally, but we have no recreation, for our work is from day to day throughout the year. I question if a man does not do as well by working five hours as by ten hours per day; he can get about as much in way of fees if he is a competent man. I find great relief in reading the literature of our profession."

*Dr. Gray.*—I agree with Dr. Smith looking at this subject in a physiological way. Who ever heard of an athlete being troubled with nervousness? But those of sedentary habits suffer from indigestion, constipation, etc., and will naturally be cross, nervous and out of fix; whenever we defy the laws of health we must suffer the consequences. We, as dentists, do not take exercise enough; we can not expect to keep ourselves in a physiological state. If a man is confined for eight hours by his profession let him have some dumb-bells or chest exerciser and get up a perspiration, then take a quick bath and rub himself vigorously; this will rest and refresh him. Many times when

I have been so nervous that I was in a quiver I would get on my horse and gallop away and after a long ride come back much refreshed.

*Dr. R. E. Taylor*—I do not believe in the electrical theory as advanced by the gentlemen who have just spoken. I do believe, however, that if a man is earnest and honest in his work and tries to please his patients, just in that proportion is this nervous strain on the system.

If you get a case where the operation is not diffiult there is no particular strain, but if it is a difficult operation and you can not get at some point you are striving to, with excavator or plugger, or you feel that the patient has but little confidence in your ability it becomes a mental strain that tells more upon dentists than upon any other professional men. Take the surgeon for comparison: Even the operation of amputating a leg or arm does not cause so great a strain as we experience in many operations about the mouth. In this case the surgeon realizes that the patient is under the influence of an anæsthetic and he works away without the strain that dentists experience in excavating a very sensitive cavity.

Dr. Berry has been referred to as a man of great endurance. I was twice associated with him in business and probably know his characteristics better than any other man. He was different from most of us in that he could lay down an instrument and inside of three minutes be asleep. I have seen him drop off to sleep with a plate in one hand and a file in the other, and after a few minutes awake thoroughly refreshed. We all know that sleep is nature's great restorer and I believe that this habit had more to do with his wonderful powers of endurance and long professional life than anything else.

